# Popular Prejudices

**Published:** 1876-01

**Authors:** E. H. Gibbs


### POPULAR PREJUDICES.

BY E. H. GIBBS, M. D.

If men were appreciated for the good they do, such names as Harvey and Jenner would be written on imperishable bronze in every civilized land.

There is, unfortunately, in human nature, a natural perversity, which the highest culture often fails to subdue. It is difficult to divest men of prejudice. They confound it with the "spirit of independence," and cling to it, and it clings to them. Like any other *disease*, it increases with the lapse of time. What they condemn, they condemn in gross. Whatever is evil in their conceit, is evil through and through. The rule admits of no "exceptions." Medicines in unskilful hands often do great harm, therefore they urge all medicines are dangerous, and should be avoided. No one but the medical man knows to what extent this foolish prejudice has taken hold of the minds of many intelligent people. While admitting that there is some reason for it, in the suffering that has been inflicted upon individuals by incompetent practitioners, it is not sufficient to justify a sweeping condemnation of doctors and drugs. There are thoughtful, studious, earnest physicians, who are devoting their lives to the good of their fellow-men, with little recompense beyond the consciousness of well doing. What would the world do to-day without the work that has been accomplished by those who have lived and died, unknown and unhonored?

The venerable Dr. Nott used to caution his students against "growing old" *mentally*. He said it was an evidence of weakness that should be appropriately treated like any other disease of the mind. The most prominent symptoms of this disease are prejudice, conceit and incredulity. These unenviable traits are in no respect so apparent as in that which relates to their bodily welfare.

The man who has no confidence in any thing, goes limping and groaning to an untimely grave, tortured to death by some disease that was entirely curable. The only thing about him to admire was his consistent obstinacy. The loss would be small did he not too often drag with him those who are compelled to be martyrs to his tyranny.

A boy had been kicked by a horse and both legs were broken and lacerated. A surgeon came to repair the damage. The *pious* mother objected to any interference, saying that if it was the Lord's will that the boy should recover, he would recover without the doctor's aid. The doctor replied, "Madam, if it is the Lord's will that you should have a good dinner to-morrow, it is not probable He will cook it for you; and if your legs were in the condition of this helpless boy's, I would obey your request and trust you to the Lord. But, impelled by motives of humanity that you are too ignorant and too bigoted to understand, I shall set these bones, though I should be compelled to break all your's to prevent your interference."

The prevailing ignorance in regard to the causes, prevention and cure of diseases is astonishing. Persons who devote their time to the work of acquiring a knowledge of the light literature of the fashionable drawing-room, entirely neglect to inform themselves in relation to those things which are of vital importance to themselves and to every living being. Medical science is advancing rapidly, but they take no note of it, and ignorantly and listlessly suffer themselves to be destroyed by diseases that are curable.

I have insisted for years that all diseases are curable, unless the constitution is broken down, or there is actual destruction or change in a vital organ. These views are being dailv confirmed by observation. During my hospital experience, dating back more than ten years, cancer was considered a fatal disease, beyond all remedies; and whatever treatment was employed was thought to be simply palliative. It is true we had reports of cases successfully treated, but these reports were not credited, because physicians then believed that all true cancerous affections were hereditary, and necessarily affected the constitution. Experience proves that this is not true in most cases.

It seems to be decided that cancer can be originated in a person, and that it is transmitted by inheritance. There is a false and a true cancer—mild and malignant. It may be said with reason that all forms of cancer are curable—some easily, others slowly. Therefore, when one has a sore which may be cancerous, or may be not, which may be a true cancer, or not—and as all forms are amenable to remedies if taken in hand very early, and we cannot tell when the boundary line between recovery and the grave is reached—it would seem rational to make a prompt effort to apply remedies which have appeared to be successful in similar cases. Cases are recorded of the spontaneous cure of cancer by Bayle and Tricod. Many eminent surgeons have reported cases remaining well after having been cured twenty years. Dr. Willard Parker has reported three cases well after having been cured of cancer twenty, twenty-two, and twenty-seven years before. He says that forty-five of his cases were caused by mental anxiety. Professor Fordyce Barker cured two cases in 1873. Dr. Wm. W. Crandall reports three cases cured, and Dr. Daniel Lewis has given seven cases successfully treated by him, one a malignant tumor on the forehead.

Several most remarkable cases of permanent cure are in and near New York, and can be seen any day. Some of them are my own patients. Information will be given in reference to these matters without charge.

